# Milk, No Thirst Quencher

**Published:** 1890-12

**Authors:** 


MILK, NO THIRST QUENCHER.—In a *brochure* lauding his thirst quenchers, an Irish provincial chemist imparts the following information:

He says, "It is a mistake to look on milk as a beverage. It is a liquid food and though it quenches the thirst for the moment, it makes it more intense after it has been in the stomach for some time, and the process of digestion has commenced.—*Ex.*

---

Potassium bromide is an efficient antidote for poisoning from iodoform.

